# Exploring the Effect of Market Conditions on Price Premiums in the Online Health Community

**DOI:** 10.3390/ijerph17041326

**Published:** 2020-02-19

**Authors:** Chaoran Li, E. Zhang, Jingti Han

**Affiliations:** 1 School of Information Management and Engineering, Shanghai University of Finance and Economics, Shanghai 200433, China; li.chaoran@163.sufe.edu.cn (C.L.); hanjt@mail.shufe.edu.cn (J.H.); 2 Institute of Fintech, Shanghai University of Finance and Economics, Shanghai 200433, China; 3 Guizhou Key Laboratory of Big Data Statistics Analysis, Guizhou University of Finance and Economics, Guiyang 550025, China

**Keywords:** e-health, online health community, online service, price premium, market conditions, stigmatized diseases, social stigma, fixed effects, I10, L10, M30

## Abstract

Online health communities allow doctors to fully use existing medical resources to serve remote patients. They broaden and diversify avenues of interaction between doctors and patients using Internet technology, which have built an online medical consultation market. In this study, the theory of supply and demand was adopted to explore how market conditions of online doctor resources impact price premiums of doctors’ online service. Then, we investigated the effect of the stigmatized diseases. We used resource supply and resource concentration to characterize the market conditions of online doctor resources and a dummy variable to categorize whether the disease is stigmatized or ordinary. After an empirical study of the dataset (including 68,945 doctors), the results indicate that: (1) the supply of online doctor resources has a significant and negative influence on price premiums; (2) compared with ordinary diseases, doctors treating stigmatized diseases can charge higher price premiums; (3) stigmatized diseases positively moderate the relationship between resource supply and price premiums; and (4) the concentration of online doctor resources has no significant influence on price premiums. Our research demonstrates that both the market conditions of online doctor resources and stigmatized diseases can impact price premiums in the online medical consultation market. The findings provide some new and insightful implications for theory and practice.

## 1. Introduction

With the development of the Internet, emerging online health communities allow doctors to fully use their spare time and medical knowledge to serve remote patients [1]. Online health communities also allow many patients to obtain online medical services for a variety of diseases through online platforms [2]. Through the Internet, online health communities can solve or alleviate medical resource shortages and doctor–patient disputes to a certain extent [1,2,3]. Communications between doctors and patients posted on online health communities contain a variety of valuable information that can help establish knowledge-based online support for online patients [4]. Most online health communities are categorized by two main functions: information search and social support [5,6]. Online health communities facilitate the rapid finding of healthcare information and professional advice [7,8]. Online health communities also reduce medical treatment costs for patients and extra returns for doctors, decreasing health disparities for society as a whole, in addition to providing other benefits [5,9,10,11]. Currently, many online health communities provide similar online services for doctors and patients, such as Good Doctor and Chunyu Doctor in China, and Quickrxrefill, MDproactive, and Livehealthonline in the USA.

Compared with doctors in physical hospitals, in online health communities, doctors have more options to choose service provision at their convenience [12]. Online medical consultation, as one of the most popular and useful service functions, is provided for patients who need professional medical diagnosis and do not visit hospitals [13]. Doctors in online health communities have the pricing power for the online medical consultation service [12]; they can set price for the online medical consultation service based on their capabilities and circumstances [14,15]. Many studies have examined factors affecting service price and returns [11,12,16], such as doctor reputation, professional title, and hospital rank. Recent studies have mainly focused on the relationships between service prices and doctors’ personal characteristics, which is necessary but insufficient. Online health communities have built an online medical consultation marketplace for doctors and patients [17,18]. In this online market, doctors with strong medical skills can obtain high service fees above the average price, to charge price premiums and receive higher returns [19,20]. In our study, the research context, Good Doctor (Haodf.com), is one of the leading and typical online health communities providing these services in China. Therefore, studying online health communities from the perspective of economics and management can provide new insights into understanding the business model of online health communities and developing online health communities. Understanding the economic factors impacting price premiums can help doctors obtain higher incomes [21,22] and help online health communities attract more and better doctors, thereby promoting the development and prosperity of such platforms.

In online health communities, online medical consultations for different medical specialties and diseases have different prices. Different types of diseases have different characteristics (e.g., severity, generality, and privacy). Existing studies show that the severity of diseases [1,23,24] and the generality of the disease [24] can impact doctors’ online services and patients’ consultation behaviors. Compared with ordinary diseases (e.g., cold, fracture, and diabetes), AIDS, hepatitis B, and psychosis are stigmatized diseases. Patients with stigmatized diseases have stronger privacy concerns than patients with ordinary diseases [25], and they are reluctant to disclose health problems due to discrimination [26]. However, regarding the stigmatized diseases, few studies have studied their influence on doctors and patients [20,27]. Therefore, it is important to study the influence of stigmatized diseases on the price premiums of online doctors in online health communities.

Doctors in online health communities are regarded as one kind of online medical resource. According to theories on economics and management, in this study, we examined whether the supply and concentration of online medical resources can impact the price premiums of online medical consultation services. We define the participation ratio of doctors as a proxy of the resource supply and the concentration ratio of online doctors as a proxy of the resource concentration to characterize the market conditions. Because patients with stigmatized diseases and corresponding doctors behave differently, we studied whether an online medical consultation service for stigmatized diseases can charge higher price premiums and how the social stigmatization of disease moderates the relationships between market conditions and price premiums of online medical consultation service.

The rest of this paper is organized as follows. We first introduce the theoretical background and hypotheses in Section 2 and then present the methodology including variables description and the estimation model in Section 3. Section 4 presents the results. Section 5 discusses the results, implications, and limitations. Finally, we conclude this research in Section 6.

## 2. Research Model and Hypotheses

### 2.1. Theory of Supply and Demand

Classical economics presents a model of the interactions among price, supply, and demand. Based on the theory of supply and demand, when demand is fixed, the larger the supply, the lower the price [28]. With increasing supply, more competitors and potential new competitors enter the market and the competition in the market increases [29]. Generally, the fiercer the competition, the less potential to achieve a profit [30]. Accordingly, Kuang et al. found that the online penetration rate positively affected the quality of doctors’ services and negatively affected the online service price in online health communities [31].

As the number of suppliers increases, markets in different industries evolve different market structures. Market structures refer to the situation and intensity of competition. The measurement of market structure usually uses the market concentration [32]. The higher the market concentration, the more monopolized the market, the higher the monopoly prices the sellers obtain. For offline primary care physicians’ services, Pauly and Satterthwaite reported that increasing the number of doctors can lead to an increase in monopoly power [33]. Then, the equilibrium price in the market increases.

In online health communities, doctors from different hospitals provide online medical consultation services related to their clinical specialty. Patients will choose a doctor from whom to buy an online medical consultation service based on their illness and physical conditions [24,34]. The whole process and businesses build a market for online medical consulting services products [17,35]. Like the e-business market, questions remain about resource supply and competition among doctors in the online medical consultation market.

### 2.2. Research Hypotheses

**Figure 1:**
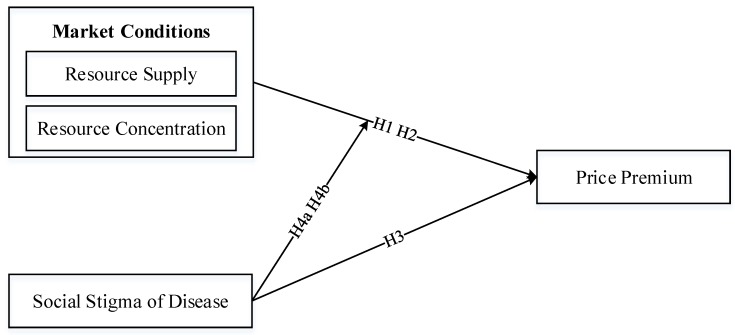
Research model.

Based on prior studies and relevant theories, we developed a research model including two factors (resource supply and resource concentration) about market conditions and one factor (social stigma) about disease characteristics to explore their effects on price premiums of doctors’ online medical consultation service in online health communities. The research model is shown in Figure 1. As shown in the model, market conditions and social stigma impact price premium, and social stigma moderates the effects of market conditions on price premium.

In online health communities, each hospital and department usually has different potential patients due to their capability and patient preferences, but the number is relatively stable. These potential patients are shared by the doctors who provide online medical consultation services [31]. In a particular hospital, some clinical departments have a high percentage of doctors who provide online medical consultation services, whereas others have a low percentage. For a particular clinical department, the percentage of doctors providing online medical consultation services in some hospitals is also different. Therefore, the supply of online doctor resources differs. Then, the competition intensity among doctors in each clinical department of the hospital is different. The fiercer the competition, the lower the profits of the providers [36], so we propose the following hypothesis:

**Hypothesis 1** **(H1).** *The supply of online doctor resources negatively impacts the price premiums of doctors’ online medical consultation services.*

In the online medical consultation market, as service providers, doctors compete with each other. This competition prompts doctors to take action in terms of price and service quality based on their capabilities and market conditions to achieve higher economic and social returns [15]. When a patient buys an online medical consultation service for a specific type of disease, the patient has to select a doctor whose clinical department is related to their disease for online consultation. For example, when a patient has a digestive disorder or a mental illness, the patient will choose a doctor from the Gastroenterology or Psychiatry departments for consultation. For a particular clinical department, some hospitals have many online doctor resources from which the patient can choose, whereas others have few resources. Then, for a particular clinical department, if a small number of hospitals have a large number of online doctors, the online doctor resources of this kind of clinical department are highly concentrated. Different clinical departments are different in terms of the concentration of online doctor resources. When the concentration of online doctor resources in a clinical department is high, a market monopoly will appear. The service providers may charge higher monopoly prices [37,38]. Therefore, we propose the following hypothesis:

**Hypothesis 2** **(H2).** *The concentration of online doctor resources positively impacts the price premiums of doctors’ online medical consultation services.*

For a variety of reasons, people may not want to disclose their health problems. Diseases have social characteristics; people with stigmatized diseases may be discriminated against and treated unfairly [26]. People with stigmatized diseases such as AIDS, hepatitis B, and psychosis have stronger privacy concerns than those with ordinary diseases [26]. For stigmatized diseases, patients have relatively few channels through which they obtain disease knowledge [20], and they are not willing to consult with friends. These patients do not wish to attend the hospital for fear of revealing personal information and embarrassment. Therefore, the diagnosis and treatment of stigmatized diseases are more difficult [20].

With online health communities, remote online medical consultation services can help patients to avoid the embarrassment of face-to-face communication with doctors and obtain a reliable and primary diagnosis; this also can reduce the likelihood that others—especially acquaintances—will find them suffering from stigmatized diseases. Online health communities can effectively help people to obtain professional medical services and avoid discrimination. Generally, the health information that an individual seeks is closely related to their individual characteristics [39]. Patients with stigmatized diseases behave differently. Patients with stigmatized diseases may prefer to use online medical consultation services and desire more direct information. In this study, we assumed that compared with ordinary diseases, patients with stigmatized diseases are more likely to accept higher service prices, and the market conditions of online doctor resources may have different influences on the service prices of doctors who treat stigmatized diseases. The specific hypotheses are as follows:

**Hypothesis 3** **(H3).** *Compared with ordinary diseases, doctors treating stigmatized diseases can get a higher price premium.*

**Hypothesis 4a** **(H4a).** *Stigmatized diseases positively moderate the relationship between resource supply and the price premiums of doctors’ online medical consultation services. Thus, for doctors treating stigmatized diseases, the resource supply has less influence on the price premiums of doctors’ online medical consultation services.*

**Hypothesis 4b** **(H4b).** *Stigmatized diseases positively moderate the relationship between resource concentration and the price premiums of doctors’ online medical consultation services. Thus, for doctors treating stigmatized diseases, the resource concentration has a stronger influence on the price premiums of doctors’ online medical consultation services.*

## 3. Materials and Methods

### 3.1. Research Context and Data Collection

**Figure 2:**
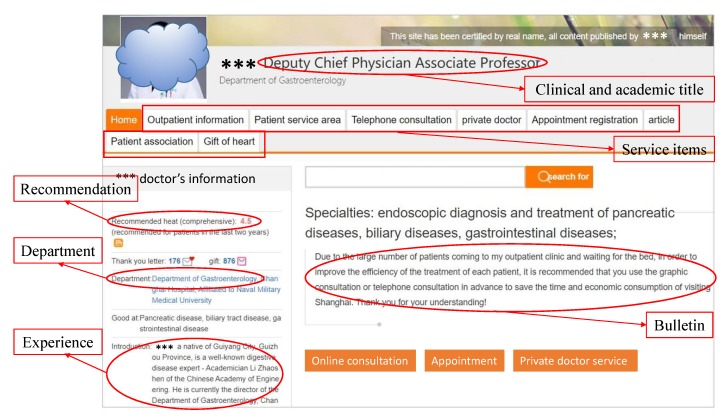
Sample of the haodf.com website.

Our research context was “Haodf online” (Haodf means “good doctor” in Chinese; www.haodf.com), which is one of the most popular and professional online health communities in China. It was founded in 2006, and currently the information of over 8000 hospitals and 500 thousand doctors is presented on this website. Haodf.com is the largest doctor–patient interaction platform in China and helps many patients by providing online services: online medical consultation, disease knowledge research, offline outpatient care appointment service, etc. Doctors registered on haodf.com have a personal homepage, which contains information such as the doctors’ background (clinic title, academic title, work experience, etc.), patients’ views, and information about online service. By this website, patients can get professional online medical services. Figure 2 shows the overview of haodf.com, for English readers’ convenience, we have translated the original Chinese version to English.

We collected data from haodf.com in July 2019, covering the public information of hospitals, departments, and doctors listed on this website. After data cleaning, we obtained a sample dataset composed of 68,945 doctors from 1000 hospitals and 31 provinces (municipalities) in mainland China.

### 3.2. Variable Measurement

The studied online medical consultation service was an online written consultation service. When doctors provide online medical consultation services in online health communities, and doctors with stronger medical skills charge a higher service price than others [12,20]. Therefore, we define the price premium as how much a doctor’s service price is above the average price, representing the doctor’s extra value. This was the dependent variable.

Each clinical department of each hospital has a different number of doctors providing online medical consultation services, so the supply of online doctor resources is different. We used the participation ratio (*PR*) to measure the supply of online doctor resources that provide online medical consultation services in each clinical department in each hospital. *PR* is equal to the number of doctors providing online medical consultation services divided by the total number of doctors in the clinical department of the hospital.

The concentration ratio (*CR*) was calculated using Equations (1) and (2) to measure the concentration of online doctor resources of each clinical department in each province. $Percentage_{phj}$ is the percentage of online doctor resources in clinical department *j* of hospital *h* in province *p*. $Number_{phj}$ is the number of doctors providing online medical consultation services in clinical department *j* of hospital *h* in province *p*. $H_{p}$ is a set of hospitals located in province *p*. $CR_{pj}$ is the concentration ratio of clinical department *j* in province *p*. $H_{pjtop}$ is a set of hospitals whose $Percentage_{phj}$ is in the top 10% given *p* and *j*. For example, in Shanghai, the *CR* of Gastroenterology is 0.477; in Jiangsu province, the *CR* of Gastroenterology is 0.353. We used *PR* and *CR* as proxies for resource supply and concentration, respectively; they were independent variables in the research model.

(1) $$Percentage_{phj}=\frac{Number_{phj}}{\sum_{h\in H_{p}}Number_{phj}}$$

(2) $$CR_{pj}=\sum_{h\in H_{pjtop}}Percentage_{phj}$$

Table 1 describes the variables included in the model. We defined social stigma as a dummy variable to categorize diseases as stigmatized or ordinary. For stigmatized diseases, patients have stronger privacy concerns and are more sensitive than patients with ordinary diseases. For example, cold, fracture, and diabetes are ordinary diseases; AIDS, hepatitis B, and psychosis are stigmatized diseases. We considered social stigma as a moderator variable and used clinic title, academic title, work experience, bulletin length, service items, recommendation, and online time as control variables in the model.

### 3.3. Estimation Model

To test the effects of market conditions on the price premiums of doctors’ online medical consultation services, we constructed the model below with multiple fixed effects. To estimate the two models in Equations (3) and (4), we used ordinary least squares (OLS), two-stage least squares (2SLS), and an endogeneity test. All the empirical models developed based on Equations (3) and (4) were estimated using STATA software version 15.0 (StataCorp LLC, College Station, TX, USA).

(3) $$\begin{array}{cc} PP_{phji}= & \beta_{0}+\beta_{1}PR_{phj}+\beta_{2}CR_{pj}+\beta_{3}SS_{j}+\beta_{4}ClinicT_{phji}+\beta_{5}AcdT_{phji}+\beta_{6}Exp_{phji}+\beta_{7}BL_{phji}\\ \; & +\beta_{8}SerItems_{phji}+\beta_{9}Rec_{phji}+\beta_{10}OnlineT_{phji}+Province_{i}+Hospital_{i}+\epsilon_{phji} \end{array}$$

where *p* indicates provinces, *h* indicates hospitals, *j* indicates clinical departments, and *i* indicates doctors. To control for unobserved heterogeneity influencing doctors’ price premiums, we employed province-level and hospital-level fixed effects to absorb differences caused by the provinces and hospitals. $Province_{i}$ indicates province fixed effects, which controls for differences across provinces such as population, economy, income, and consumption. $Hospital_{i}$ indicates hospital fixed effects, which controls for differences across hospitals such as transportation, service quality, and hospital level. Fixed effects models are often used to remove common trends over individuals in econometrics [40,41,42,43].

Then, we formulated Equation (4) by adding interactions to examine the moderating effects of social stigma.

(4) $$\begin{array}{cc} PP_{phji}= & \beta_{0}+\beta_{1}PR_{phj}+\beta_{2}CR_{pj}+\beta_{3}SS_{j}+\beta_{4}SS_{j}\times PR_{phj}+\beta_{5}SS_{j}\times CR_{pj}+\beta_{6}ClinicT_{phji}\\ & +\beta_{7}AcdT_{phji}+\beta_{8}Exp_{phji}+\beta_{9}BL_{phji}+\beta_{10}SerItems_{phji}+\beta_{11}Rec_{phji}+\beta_{12}OnlineT_{phji}\\ & +Province_{i}+Hospital_{i}+\epsilon_{phji} \end{array}$$

Even though we used the multiple fixed effects model to avoid missing unobserved variables, endogeneity may still have been caused by other reasons, which would bias the estimation results. Therefore, we employed the two instrumental variables listed below and the 2SLS method to test the hypotheses. The two variables are exogenous, which cannot be impacted by price premiums.

- *Offline concentration ratio* (*OfflineCR*): In physical hospitals, each clinical department has many offline doctors. Some doctors provide online medical consultation services (OMCSs) in online health communities (OHCs), others do not. Online doctor resources are obtained from offline doctor resources. So, *OfflineCR* measures the concentration of offline doctor resources, whereas *CR* measures online doctor resources.
- *The average participation ratio* (*PRmean*): As shown in Equation (5), given province *p* and clinical department *j*, this is the sum of the participation ratio ($PR_{phj}$) divided by the number of hospitals that provide the OMCS associated with clinical department *j* ($|H_{pj}|$). $H_{pj}$ is a set of hospitals that provide the OMCS associated with clinical department *j* in province *p*.
  (5) $$PRmean_{pj}=\frac{\sum_{h\in H_{pj}}PR_{phj}}{|H_{pj}|}$$

## 4. Results

### 4.1. Descriptive Statistics and Correlations

Table 2 lists the descriptive statistics and correlations (Pearson correlation coefficients). Independent variables were found to have significant relationships with the dependent variable. The correlations among independent variables and control variables were low. When estimating the models, the variance inflation factor (VIF) of variables was less than 2.0, so the multicollinearity problem was not serious.

### 4.2. Empirical Results

Table 3 provides the empirical results. Model 1 was the baseline. Its adjusted determination coefficient ($R^{2}$) was 0.290, but it missed unobserved information about province and hospital, which led to severely biased estimation. As an improvement, Model 2 employed province-level and hospital-level fixed effects to control for differences caused by these two features; its adjusted $R^{2}$ increased to 0.385. In model 2, the coefficient of *PR* was negative and significant ($\beta_{1}$ = −3.756, *p* < 0.01), indicating that the supply of online doctor resources negatively impacted doctors’ price premiums. *CR* had a positive and significant coefficient ($\beta_{2}$ = 2.649, *p* < 0.01), indicating that the concentration of online doctor resources positively impacted doctors’ price premiums. The coefficient of *SS* was positive and significant ($\beta_{3}$ = 4.675, *p* < 0.01), indicating that social stigma positively impacted doctors’ price premiums.

Model 3 shows the estimation results using the 2SLS method with instrumental variables. The weak identification test showed that there was no weak instrument problem. The Durbin–Wu–Haussmann (DWH) test with heteroscedasticity robustness showed that there was a significant difference between the estimation results of the 2SLS and OLS methods ($\chi^{2}$ = 32.075, *p* < 0.05). Therefore, consistent estimation is possible using the 2SLS method. In Model 3, *PR* had a negative and significant coefficient ($\beta_{1}$ = −7.703, *p* < 0.01), which means that the supply of online doctor resources negatively impacted doctors’ price premiums. Hypothesis 1 is supported. The coefficient of *CR* was not significant ($\beta_{2}$ = 1.472, *p* > 0.05), so Hypothesis 2 is not supported. The coefficient of *SS* was positive and significant ($\beta_{3}$ = 4.780, *p* < 0.01), demonstrating that doctors treating stigmatized diseases can get a higher price premium compared with doctors treating ordinary diseases. Hypothesis 3 is supported.

**Figure 3:**
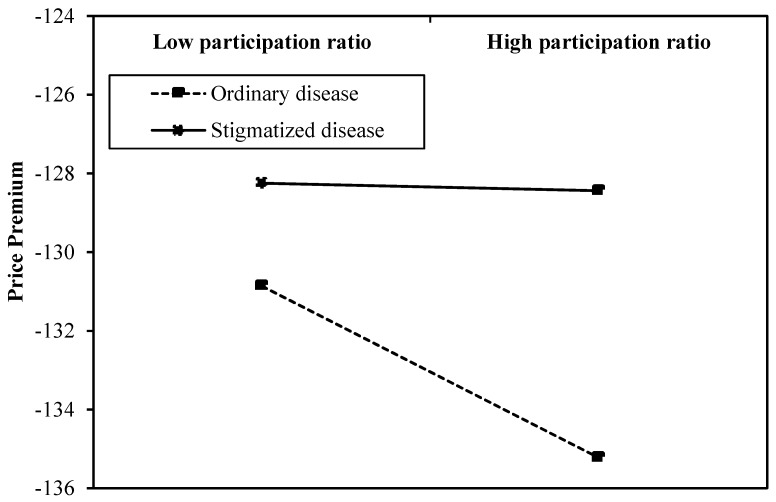
The moderating effect of social stigma.

Model 3 shows that *CR* did not have a significant influence on price premiums; therefore, Model 4 only examined the moderating effects of social stigma on the relationship between *PR* and price premiums. The result showed that social stigma positively moderated the relationship between *PR* and price premiums ($\beta_{4}=8.943$, *p* < 0.01). For doctors treating stigmatized diseases, the resource supply had less of an influence on the price premiums of doctors’ online medical consultation service. So, Hypothesis 4a is supported, and Hypothesis 4b could not be tested. To develop a more nuanced understanding, we performed simple slope tests and plotted the moderating effect in Figure 3. As the participation ratio increased, the price premiums of doctors’ online medical consultation service decreased, regardless of doctors treating stigmatized or ordinary diseases. However, for doctors treating stigmatized diseases, the decline in their price premiums was lower than those of doctors treating ordinary diseases. Therefore, for stigmatized diseases, the supply of online doctor resources had a less-negative effect on the price premiums of doctors’ online medical consultation services.

### 4.3. Robustness Check

We used two approaches to check the robustness of the above results. First, the doctor’s service price minus average service price of the doctor’s located province is defined as provincial price premium (*PPP*), which we used to replace the price premium. In Models 5 and 7, the results are consistent with the results of Models 3 and 4. Second, we used the generalized method of moments (GMM) to replace the 2SLS method to estimate the models. Models 6 and 7 show that the estimation results were similar to Models 3 and 4. Hereto, our results seem to be quite robust.

## 5. Discussion and Implications

### 5.1. Results Analysis

The empirical results showed that the participation ratio of doctors providing online medical consultation service in the clinical department negatively impacted the doctors’ price premiums. As more doctors provide online medical consultation services, the supply of online doctor resources will increase, providing more supply and choice for patients. Meanwhile, competition among doctors will increase, so price premiums will decrease. Finally, the increasing supply of online doctor resources leads to lower price premiums.

We found that the concentration ratio of online doctor resources did not significantly influence the price premium of doctors’ online medical consultation service. We investigated the reason for this finding. Online doctor resources are from offline doctors in hospitals. The hospitals are planned and established by the government according to the existing doctor resources and patients’ needs within the region as well as regional development plans. Therefore, even if some excellent hospitals have relatively large amounts of doctor resources, no severe imbalance or monopoly of doctor resources exists within any region. In the online medical consultation market, hospitals have not formed alliances; they all operate independently, so no monopoly group has formed. The hospital does not intervene in the pricing of doctors’ services. As an independent individual, doctors have not realized a monopoly on resources and have not formed group wisdom. As an e-business market, competition is the primary trend instead of monopoly. So, the price premiums are not significantly influenced due to the different resources’ holdings.

Finally, doctors providing online medical consultation services for stigmatized diseases can get higher price premiums. Stigmatized diseases positively moderated the relationship between the supply of online doctor resources and the price premiums of doctors’ online medical consultation services. Compared with ordinary diseases, patients with stigmatized diseases are willing to pay higher service fees to receive direct and professional information. For doctors treating stigmatized diseases, the supply of online doctor resources had less influence on the price premiums of doctors’ online medical consultation services. Considering this, sharing more knowledge about stigmatized diseases on online platforms and reducing disease discrimination are necessary for promoting public health. The online health community is a useful channel for disseminating disease knowledge and providing patients with disease pre-diagnosis.

### 5.2. Implications

Our findings provide the following theoretical contributions. Firstly, we regarded online health communities as an online market and expanded the scope of existing online health community research by incorporating theories in economics and management. Secondly, we investigated how the market conditions of online doctor resources impact doctors’ price premiums in the online market. Prior studies mainly focused on the relationships between doctors’ characteristics and prices. We bring a new perspective to current research. Thirdly, we contribute to the research about the psychology of diseases. Due to patients’ concerns about revealing health information and the feeling of shame, patients with stigmatized diseases tolerate higher prices. Fourthly, we used a multiple fixed effects model to absorb differences caused by province and hospital doctors and to improve the models used in prior studies of online health communities.

The findings also provide practical insights. Firstly, the findings of the influence of price premiums are valuable for managers of online health communities when planning commercial strategies to develop these kinds of online platforms. Well-developed online health communities will attract more doctors and patients to participate in online platforms. This research could help doctors to understand the rules of price in the online medical consultation market. Secondly, for society and the public, this study revealed that stigmatized diseases are inevitable, the anxiety about stigmatized diseases affects patients when seeking disease and medical information, and they are willing to pay higher medical costs to maintain privacy. Therefore, society and the public should treat stigmatized diseases correctly and aggressively rather than avoiding them and keeping silent.

### 5.3. Limitations and Future Research

This study has several limitations, and we have recommendations for future research directions. Firstly, we used a cross-sectional analysis, so we could not observe the dynamic changes over time; future research could adopt longitudinal data or panel data to improve the current research. Secondly, we only studied one context (haodf.com). Although it is one of the largest online health communities in China, our findings may lack generalizability. Our results need to be validated in other online health communities. Thirdly, regarding the market conditions, we will try to measure them according to the patient numbers in online health communities.

## 6. Conclusions

Our study explored the effect of market conditions of online doctor resources on the price premiums of doctors’ online medical consultation services in online health communities and investigated the direct and moderating effects of stigmatized diseases. We developed a model with multiple fixed effects to test our hypotheses by using a 2SLS method. The empirical results indicate that the supply of online doctor resources has a significant and negative influence on the price premiums of doctors’ online medical consultation services. Compared with ordinary diseases, doctors treating stigmatized diseases can get a higher price premium. Stigmatized diseases positively moderate the relationship between resource supply and the price premiums of doctors’ online medical consultation service. Thus, for doctors treating stigmatized diseases, the resource supply has less influence on the price premiums of doctors’ online medical consultation service. Our results contribute to the studies about online health communities and provide suggestions to managers of online health communities, doctors, society, and the public.

## Figures and Tables

**Table 1:** Variables description.

Variables	Description
**Dependent variable**	
Price premium (*PP*)	Doctor’s service price minus national average service price.
**Independent variables**	
Participation ratio (*PR*)	The percentage of doctors providing online medical consultation services in each clinical department in each hospital. Proxy for resource supply.
Concentration ratio (*CR*)	An index of market structure to measure the concentration of online doctor resources in each clinical department in each province. Proxy for resource concentration.
**Moderator variable**	
Social stigma (*SS*)	Dummy variable to show whether the disease is a stigmatized disease. SS = 1 if it is a stigmatized disease, SS = 0 if it is an ordinary disease.
**Control variables**	
Clinic title (*ClinicT*)	Clinic title is certified by the national agency with uniform standards, four stages (Chief Physician, Deputy Chief Physician, Attending Physician, and Resident). ClinicT = 1 if a doctor is Chief or Deputy Chief Physician, and 0 otherwise.
Academic title (*AcdT*)	The academic title is certified by the educational system, four stages (Professor, Associate Professor, Lecturer, and Assistant). AcdT = 1 if a doctor is a Professor or Associate Professor, and 0 otherwise.
Work Experience (*Exp*)	Character length of work experience.
Bulletin length (*BL*)	Character length of the bulletin.
Service items (*SerItems*)	The number of service functions provided by a doctor.
Recommendation (*Rec*)	The recommendation level displayed on the homepage.
Online time (*OnlineT*)	The number of months since the doctor first joined the online health community.
**Others**	
Province	The province (municipality) where a doctor works.
Hospital	The hospital to which a doctor belongs.
Department	The clinical department to which a doctor belongs.

**Table 2:** Description and correlation.

Variables	Min	Max	Mean	Std Err	1	2	3	4	5	6	7	8	9	10	11
1. PP	−19	976	0	26.62	1										
2. PR	0.005	1	0.407	0.233	−0.051 ***	1									
3. CR	0.111	1	0.383	0.128	0.066 ***	0.056 ***	1								
4. SS	0	1	0.115	0.319	0.074 ***	0.029 ***	0.032 ***	1							
5. ClinicT	0	1	0.467	0.499	0.238 ***	−0.068 ***	−0.014 ***	0.025 ***	1						
6. AcdT	0	1	0.198	0.398	0.236 ***	−0.073 ***	0.007 *	0.004	0.508 ***	1					
7. Exp	0	3914	113.4	139.7	0.304 ***	−0.050 ***	0.020 ***	−0.001	0.490 ***	0.478 ***	1				
8. BL	0	14,548	21.55	74.32	0.118 ***	0	0.016 ***	0.021 ***	0.108 ***	0.126 ***	0.195 ***	1			
9. SerItems	0	9	6.559	1.119	0.339 ***	0.014 ***	0.048 ***	0.067 ***	0.318 ***	0.265 ***	0.384 ***	0.206 ***	1		
10. Rec	0	5	3.72	0.295	0.517 ***	−0.035 ***	0.100 ***	0.048 ***	0.307 ***	0.337 ***	0.398 ***	0.167 ***	0.492 ***	1	
11. OnlineT	0	136	46.13	35.82	0.212 ***	−0.063 ***	0.018 ***	0.003	0.382 ***	0.388 ***	0.420 ***	0.177 ***	0.346 ***	0.245 ***	1

Note: * *p* < 0.10; *** *p* < 0.01.

**Table 3:** Model results.

Variable	Model 1	Model 2	Model 3	Model 4	Model 5	Model 6	Model 7	Model 8
*PR*	−3.512 ***	−3.756 ***	−7.703 ***	−9.355 ***	−7.703 ***	−7.703 ***	−9.355 ***	−9.355 ***
	(0.419)	(0.500)	(0.830)	(0.835)	(0.830)	(0.830)	(0.835)	(0.835)
*CR*	3.793 ***	2.649 ***	1.472	1.297	1.472	1.472	1.297	1.297
	(0.715)	(0.809)	( 1.295)	(1.289)	(1.295)	(1.295)	(1.289)	(1.289)
*SS*	4.042 ***	4.675 ***	4.780 ***	4.701 ***	4.780 ***	4.780 ***	4.701 ***	4.701 ***
	(0.325)	(0.345)	(0.340)	(0.338)	(0.340)	(0.340)	(0.338)	(0.338)
*SS×PR*				8.943 ***			8.943 ***	8.943 ***
				(2.535)			(2.535)	(2.535)
*Control variables*	Yes	Yes	Yes	Yes	Yes	Yes	Yes	Yes
Province fixed effect	No	Yes	Yes	Yes	Yes	Yes	Yes	Yes
Hospital fixed effect	No	Yes	Yes	Yes	Yes	Yes	Yes	Yes
Observations	68,945	68,945	68,945	68,945	68,945	68,945	68,945	68,945
Adjusted $R^{2}$	0.290	0.385	0.384	0.384	0.384	0.384	0.324	0.384
Weak identification test			6859.229	3898.806	6859.229	6859.229	3898.806	3898.806
Hansen J-statistics			0.000	0.000	0.000	0.000	0.000	0.000
Endogeneity test			32.075	42.141	32.075	32.075	42.141	42.141
*p*-Value			0.000	0.000	0.000	0.000	0.000	0.000

Notes: Robust standard errors in parentheses. *** *p* < 0.01.
